# Mechanical Stability of Amorphous Silicon Thin-Film Devices on Polyimide for Flexible Sensor Platforms

**DOI:** 10.3390/s26031026

**Published:** 2026-02-04

**Authors:** Giulia Petrucci, Fabio Cappelli, Martina Baldini, Francesca Costantini, Augusto Nascetti, Giampiero de Cesare, Domenico Caputo, Nicola Lovecchio

**Affiliations:** 1 Department of Information Engineering, Electronics and Telecommunications, Sapienza University of Rome, Via Eudossiana 18, 00184 Rome, Italy; g.petrucci@uniroma1.it (G.P.); fabio.cappelli@uniroma1.it (F.C.); baldini.1637322@studenti.uniroma1.it (M.B.); giampiero.decesare@uniroma1.it (G.d.C.); domenico.caputo@uniroma1.it (D.C.); 2 School of Aerospace Engineering, Sapienza University of Rome, Via Salaria 851, 00138 Rome, Italy; augusto.nascetti@uniroma1.it; 3 Research Centre for Plant Protection and Certification, Council for Agricultural Research and Economics (CREA-DC), Via Carlo Giuseppe Bertero 22, 00156 Rome, Italy; francesca.costantini@crea.gov.it

**Keywords:** hydrogenated amorphous silicon sensors, flexible electronics, polyimide (Kapton^®^), p-i-n diode, p-i-n sensor, mechanical bending, wearable sensors

## Abstract

Hydrogenated amorphous silicon (a-Si:H) is a mature thin-film technology for large-area devices and thin-film sensors, and its low-temperature growth via Plasma-Enhanced Chemical Vapor Deposition (PECVD) makes it particularly suitable for biomedical flexible and wearable platforms. However, the reliable integration of a-Si:H sensors on polymer substrates requires a quantitative assessment of their electrical stability under mechanical stress, since bending-induced variations may affect sensor accuracy. In this work, we provide a quantitative, direction-dependent evaluation of the static-bending robustness of both single-doped a-Si:H layers and complete p-i-n junction stacks on polyimide (Kapton^®^), thereby linking material-level strain sensitivity to device-level functionality. First, n- and p-doped a-Si:H layers were deposited on 50 µm thick Kapton^®^ and then structured as two-terminal thin-film resistors to enable resistivity extraction under bending conditions. Electrical measurements were performed on multiple samples, with the current path oriented either parallel (longitudinal) or perpendicular (transverse) to the bending axis, and resistance profiles were determined as a function of bending radius. While n-type layers exhibited limited and mostly gradual variations, p-type layers showed a stronger sensitivity to mechanical stress, with a critical-radius behavior under transverse bending and a more progressive evolution in the longitudinal one. This directional response identifies a practical bending condition under which doped layers, particularly p-type films, are more susceptible to strain-induced degradation. Subsequently, a linear array of a-Si:H p-i-n sensors was fabricated on Kapton^®^ substrates with two different thicknesses (25 and 50 µm thick) and characterized under identical bending conditions. Despite the increased strain sensitivity observed in the single-layers, the p-i-n diodes preserved their rectifying behavior down to the smallest radius tested. Indeed, across the investigated radii, the reverse current at −0.5 V remained consistent, confirming stable junction operation under bending. Only minor differences, related to substrate thickness, were observed in the reverse current and in the high-injection regime. Overall, these results demonstrate the mechanical robustness of stacked a-Si:H junctions on polyimide and support their use as sensors for wearable biosensing architectures. By establishing a quantitative, orientation-aware stability benchmark under static bending, this study supports the design of reliable a-Si:H flexible sensor platforms for curved and wearable surfaces.

## 1. Introduction

In recent years, interest in flexible substrates has significantly grown, as they have emerged as promising platforms for next-generation sensing and biosensing technologies, particularly in applications where conformability, low weight, and mechanical robustness are essential [1,2,3,4,5,6]. These substrates, primarily made from polymeric materials, offer several advantages over rigid counterparts, as they are low-cost, lightweight, and durable, while also being capable of bending, stretching, and conforming to irregular surfaces. Their mechanical flexibility is particularly relevant for wearable devices, such as skin-contact electronics, where thin-film devices must maintain their functionality even under repeated deformation. In this context, thin-film devices are especially appealing because they can provide lightweight, conformable and large-area components; however, their electrical characteristics may be affected by mechanically induced variations, potentially resulting in signal drift and reduced reproducibility [7,8,9,10].

Flexible electronics is rapidly expanding because polymer substrates enable lightweight, conformable devices for wearables and beyond, spanning healthcare, energy harvesting, and consumer electronics [11,12,13,14,15,16,17,18,19]. These flexible platforms can be integrated into fabrics or accessories for real-time monitoring of physiological parameters [20,21,22,23,24,25,26,27,28], and they also support foldable photovoltaics for portable power generation on unconventional surfaces [29,30,31,32,33,34]. At the same time, the conformability and biocompatibility of polymers such as polyimides and polyesters make them well suited for bioelectronic, implantable, and environmental devices [35,36,37].

Despite the aforementioned advantages, the integration of sensors and active devices onto these substrates remains challenging. Many semiconductor materials require high-temperature deposition processes, which may thermally degrade or deform the underlying polymers. Moreover, devices must maintain stable and reproducible performance under mechanical stress, including bending or twisting, demanding materials and fabrication methods that can endure these conditions. Therefore, assessing mechanical stability under deformation is crucial for developing reliable, flexible and wearable systems [38,39,40]. In addition, flexible thin-film devices may suffer from strain-sensitive interfaces and contacts, and their performance can be influenced by environmental factors (e.g., moisture), which may require appropriate encapsulation strategies depending on the target application.

As a result, both organic and inorganic semiconductors have been explored. Organics are compatible with polymer substrates but are often limited in stability and performance [41,42,43], whereas inorganics offer higher performance at the cost of more demanding processing.

Among various inorganic semiconductors, hydrogenated amorphous silicon (a-Si:H) remains an attractive candidate for thin-film flexible electronics due to its unique properties. Indeed, its ability to be deposited over large areas at low temperatures makes it compatible with heat-sensitive polymer substrates [44,45,46,47,48,49]. Additionally, the incorporation of hydrogen into the amorphous matrix effectively passivates dangling bonds, enhancing both its electronic properties and stability [50,51,52].

The integration of a-Si:H with flexibles has enabled notable advancements across various applications. For instance, thin-film transistors (TFTs) have been successfully fabricated on flexible polyimide [53,54,55,56], where they maintain stable performance under mechanical stress, demonstrating the feasibility of large-area flexible electronics for applications such as imaging systems.

In photovoltaics, flexible solar cells based on a-Si:H have shown the potential for lightweight and adaptable energy systems [57,58,59,60]. Moreover, flexible position-sensitive detectors have been developed by depositing a-Si:H onto polyimide substrates [61]. These devices offer improved performance and flexibility, making them suitable for various sensing applications.

While a-Si:H p-i-n diodes are well established in optoelectronic and photovoltaic applications, their use in flexible electronics has received comparatively less attention. Most bending-related studies have focused on TFTs [62,63,64,65], leaving the impact of mechanical strain on p-i-n diodes largely unexplored. To date, few studies have investigated how bending-induced strain affects the electrical behavior of complete p-i-n structures, particularly under realistic mechanical deformation scenarios [66]. Moreover, a-Si:H p-i-n devices exhibit temperature-dependent electrical characteristics, making them suitable as thin-film temperature sensors [67,68,69,70,71,72]. However, integrating them into flexible or wearable systems requires first assessing their mechanical resilience, since bending-induced strain can impact material properties, interface quality, and contact behavior, potentially altering key electrical parameters.

In this work, we investigated the electrical response of a-Si:H p-i-n diodes fabricated on Kapton^®^ substrates under static mechanical bending. The aim was to evaluate the influence of mechanical stress on device performance as a foundational step toward their use in flexible biosensing applications. Such sensors could be integrated into wearable health monitoring devices, smart textiles, or embedded diagnostic platforms, where flexible and conformable sensing platforms are essential. Wearable operation can involve both static curvature (conformability to a surface) and dynamic deformation (fatigue during motion); accordingly, static bending provides a first quantitative benchmark, whereas cyclic/fatigue loading is relevant for lifetime assessment and it is beyond the scope of the present experimental study. Indeed, the novelty of this work consists in linking the material-level analysis of doped a-Si:H films to the device-level robustness of complete p-i-n diodes under identical static bending conditions, with an explicit comparison between longitudinal and transverse configurations.

The experimental study was conducted in two main stages: first, the electrical response under bending of single-layer p- and n-doped a-Si:H films was characterized to assess their individual mechanical stability. These layers were subjected to controlled bending across a range of curvatures in both longitudinal and transverse directions, allowing the evaluation of anisotropic responses and the identification of critical failure thresholds. Changes in current–voltage (IV) characteristics, conductivity and resistivity were monitored throughout.

This preliminary analysis provides a quantitative framework to interpret how strain affects the electrical response of the doped layers prior to integration into full device stacks. Then, complete p-i-n diodes were fabricated and evaluated under the same conditions. In addition, by testing devices fabricated on two Kapton^®^ thicknesses, we address a practical design variable for flexible platforms. The structure of this paper follows this progression, starting with materials-level analysis and concluding with device-level performance under mechanical stress.

Overall, the study provides a quantitative basis to support the use of stacked a-Si:H junctions on polyimide as mechanically robust building blocks for flexible sensing architectures.

## 2. Materials and Methods

### 2.1. Substrate Selection

Primarily, we reviewed flexible substrates commonly employed in electronic applications to identify the most suitable material for the integration of a-Si:H devices. The selection of an appropriate flexible substrate is a critical design step in the development of mechanically robust thin-film devices. Indeed, the substrate must provide mechanical compliance while maintaining dimensional and chemical stability throughout both fabrication and operation. Key requirements include high thermal stability, low moisture permeability, chemical resistance, mechanical durability under strain, and compatibility with standard microfabrication processes. In particular, mechanical integrity should be preserved under repeated bending and rolling cycles, without inducing cracks, delamination or strain-related degradation in the active layers.

Among the available materials, polyimide (PI), commercially known as Kapton^®^ (DuPont, Wilmington, DE, USA), stands out for its excellent thermal stability, preserving its mechanical properties up to 400 °C [73]. This ensures its compatibility with Plasma-Enhanced Chemical Vapor Deposition (PECVD), the a-Si:H standard growth technique, which typically operates at 250 °C [44,69].

Kapton^®^ also exhibits chemical inertness toward common solvents (e.g., isopropyl alcohol, acetone, toluene) and acids (e.g., hydrofluoric, sulfuric, nitric), making it appropriate for subtractive and additive thin-film fabrication techniques. Moreover, its low coefficient of thermal expansion (CTE), combined with a smooth and planar surface that ensures good adhesion during thin-film growth, makes it a standard in flexible electronics [74,75,76,77].

In contrast, polyethylene terephthalate (PET) and polyethylene naphthalate (PEN) offer higher flexibility and optical transparency but exhibit limited thermal stability. PET, with a glass transition temperature (Tg) of ~70–80 °C, undergoes deformation under moderate thermal loads, thereby restricting its use in standard PECVD processes [78]. PEN presents a slightly improved Tg (~120 °C), yet it remains inadequate for high-temperature processing [78].

Nylon has recently been investigated for its ferroelectric properties and potential use in flexible and transparent electronics; however, it lacks sufficient thermal and chemical stability for microelectronic integration [79,80].

Considering all requirements, Kapton^®^ emerges as the most suitable substrate for flexible a-Si:H-based devices intended for sensor applications under bending stress.

### 2.2. Materials Context and Technology Comparison

To position the proposed a-Si:H devices on polyimide within the broader landscape of flexible sensor technologies, Table 1 provides a qualitative comparison with representative thin-film material platforms. The comparison highlights process compatibility with polymer substrates, key strengths for flexible sensing, and common limitations relevant to wearable and conformable operation.

### 2.3. Fabrication of Doped a-Si:H Layers

The study began with the characterization of the electrical properties of single-doped a-Si:H layers under mechanical bending. This preliminary investigation aimed to evaluate the mechanical and electrical stability of n-type and p-type a-Si:H films prior to the fabrication of whole p-i-n devices. The intrinsic a-Si:H layer was not characterized independently, as its very high resistivity prevents meaningful IV measurements under moderate bias conditions. Its contribution to device behavior becomes significant only within complete multilayer structures, where carrier transport is enabled by the adjacent doped layers.

**Figure 1:**
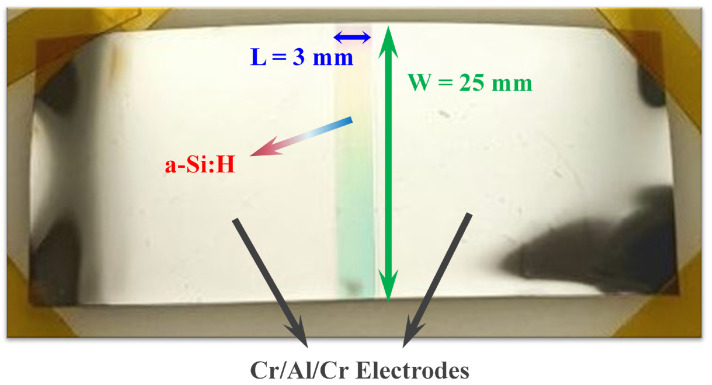
Photograph of a fabricated single-layer test structure on Kapton^®^ used for bending measurements. The sample consists of a uniformly deposited doped a-Si:H film contacted by a Cr/Al/Cr metal stack deposited through a 3 mm wide physical mask, defining a 25 × 3 mm_2_ conduction path for two-terminal IV measurements.

The doped a-Si:H layers were deposited uniformly over 50 µm thick Kapton^®^ substrates (DuPont™ Kapton^®^ HN grade, Wilmington, DE, USA) by the PECVD technique. Subsequently, a 65/350/ 65 nm thick Cr/Al/Cr metal stack was deposited through a 3 mm wide polyimide physical mask by thermal evaporation to form the electric contacts. The contact geometry was designed to delimit a conduction path of 25 × 3 mm^2^ (Figure 1), enabling standard two-terminal current–voltage measurements. The thickness of the a-Si:H layer was 400 nm for the p-type and 155 nm for the n-type samples, corresponding in both cases to a 15 min deposition under the process conditions reported in [50].

In particular, a total of four initial samples were fabricated (two p-type and two n-type), each deposited on a 9 × 9 cm^2^ Kapton^®^ substrate. After metal deposition, each 9 × 9 cm^2^ substrate was cut into three smaller specimens whose geometry is shown in Figure 1. This procedure yielded six n-type and six p-type samples, which were subsequently employed for electrical characterization under bending conditions.

### 2.4. Fabrication of a-Si:H p-i-n Linear Array

To evaluate the performance of a-Si:H junctions, a linear array of p-i-n diodes was fabricated on two Kapton^®^ substrates (DuPont™ Kapton^®^ HN grade, Wilmington, DE, USA) with different thicknesses (25 µm and 50 µm) using standard microfabrication techniques [69].

The device consists of 32 diodes, each with an active area of 2 × 2 mm^2^, featuring an individual bottom electrode and a shared top contact. Of these, 30 diodes are arranged in a 6 × 5 matrix, while two additional units are positioned outside the array. The bottom electrode, made of a 45/200/45 nm thick Cr/Al/Cr stack, was deposited via thermal evaporation. Subsequently, an a-Si:H p-type/intrinsic/n-type junction with a thickness of 10/400/30 nm was grown by PECVD. To protect the junction and ensure good ohmic contact at the top interface, the a-Si:H stack was coated with a 50 nm chromium layer by thermal evaporation. This step was followed by the deposition of a negative photoresist (SU-8) layer, patterned to define via holes for inter-layer electrical connections. Finally, a titanium–tungsten (Ti-W) alloy was deposited via sputtering to form the shared top electrode.

From an electrical interfacing standpoint, each diode is addressed through its dedicated bottom electrode, while a shared top electrode is used for probing, enabling consistent access across the array. This layout simplifies the electrical connections during bending tests and supports reliable device-to-device comparison.

**Figure 2:**
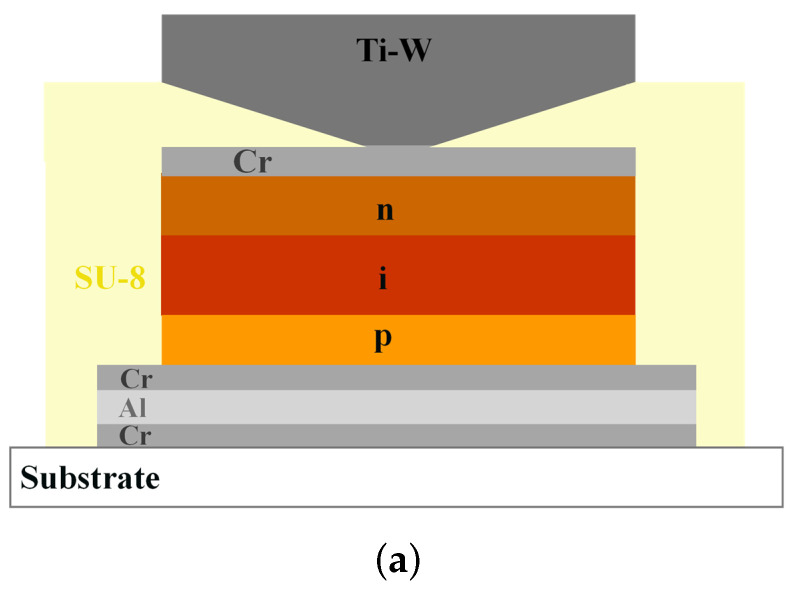

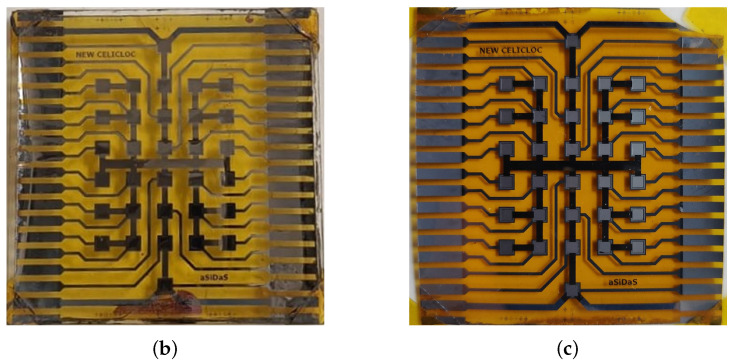
(**a**) Schematic cross-section of a fabricated diode on Kapton^®^, showing the layer stack and contacting scheme: Cr/Al/Cr bottom electrode; a-Si:H p/i/n stack; Cr capping layer; SU-8 insulating layer with via holes; Ti-W shared top electrode. Picture of the diodes array fabricated on 25 (**b**) and 50 (**c**) µm thick Kapton^®^ substrates.

The schematic cross-section of the fabricated devices is shown in Figure 2a, while pictures of the samples fabricated on the 25 and 50 µm thick Kapton^®^ substrates are presented in Figure 2b and Figure 2c, respectively.

### 2.5. Electrical Characterization of Doped Layers Under Bending

To evaluate the mechanical stability of individual doped layers, we investigated the electrical behavior of n-type and p-type a-Si:H films under mechanical bending. All measurements were performed under laboratory ambient conditions, i.e., at 25 °C and 50% relative humidity. All substrates were subjected to static bending using cylindrical supports with different radii, whose values are listed in Table 2. Since the mechanical deformation experienced by the active layers depends not only on the bending radius but also on the substrate thickness, a first-order estimate of the tensile strain at the top Kapton^®^ surface is also provided for both 25 and 50 µm thick samples.

During the tests, each sample was conformed to the cylindrical support to impose the controlled curvature, with the active a-Si:H layer located on the outer (tensile) side of the bend, consistently with the strain estimate reported in Table 2. In particular, the surface strain values ε were estimated using the relation ε≈t/(2R), where *t* is the Kapton^®^ substrate thickness and *R* is the bending radius. This approximation assumes the neutral axis to be located near the mid-plane of the polymer and neglects the contribution of the thin-film stack thickness, which is several orders of magnitude smaller than *t*. As a result, for a given bending radius, the 50 µm thick substrate experiences approximately twice the surface strain of the 25 µm thick one.

For each sample and bending radius, after an initial baseline IV measurement in the flat configuration, the experimental procedure involved two steps:

1. An IV measurement under bending;
2. A second IV measurement in the flat state, immediately after releasing the sample from the bent condition.

This procedure allowed reversible effects to be distinguished from permanent electrical changes, which may be associated with irreversible microstructural modifications in the thin films and/or at the metal/a-Si:H/substrate interfaces.

Current–voltage measurements were carried out in two orthogonal directions:

- Transverse: with current flow perpendicular to the bending axis;
- Longitudinal: with current flow parallel to the bending axis.

**Figure 3:**
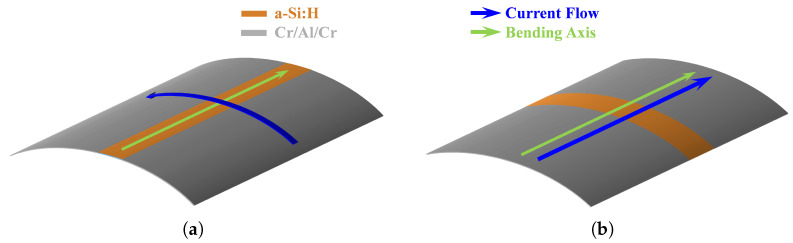
Current flow directions: (**a**) transverse; (**b**) longitudinal.

In Figure 3, the two different configurations are visible.

### 2.6. Electrical Characterization of p-i-n Device Under Bending

For the complete devices, IV measurements were recorded for each bending configuration, beginning with the unbent state and proceeding to progressively smaller bending radii. Measurements were carried out under the same laboratory ambient conditions as before (25 °C, 50% relative humidity). The experimental procedure was the same as the one used to evaluate the single-doped layers, allowing the identification of any permanent changes in the device characteristics and the detection of potential performance degradation or failure resulting from mechanical deformation. In particular, for each bending radius, IV measurements were acquired both under bending and immediately after releasing the sample to the flat state, allowing reversible and permanent effects to be distinguished.

To evaluate device integrity under bending, we track two electrical signatures: (i) preservation of rectifying IV behavior, and (ii) stability of the reverse current at a fixed reverse bias (−0.5 V), which serves as a repeatable metric for comparing different bending radii and configurations. These parameters enable quantification of bending-induced changes while maintaining a consistent test protocol across samples.

## 3. Results and Discussion

### 3.1. Electrical Response of n- and p-Doped Layers

The electrical response of the fabricated single-layer samples was evaluated under static mechanical bending. As previously described, six samples were obtained for each doping type. For each series, three samples were subjected to transverse bending and three to longitudinal bending, in order to investigate the influence of stress direction on device behavior and to ensure statistical significance. The three samples used for each bending configuration were obtained from the same substrate and exhibited comparable initial resistance values. This allowed us to study the bending behavior of samples under similar initial conditions.

#### 3.1.1. n-Type a-Si:H Layer

**Figure 4:**
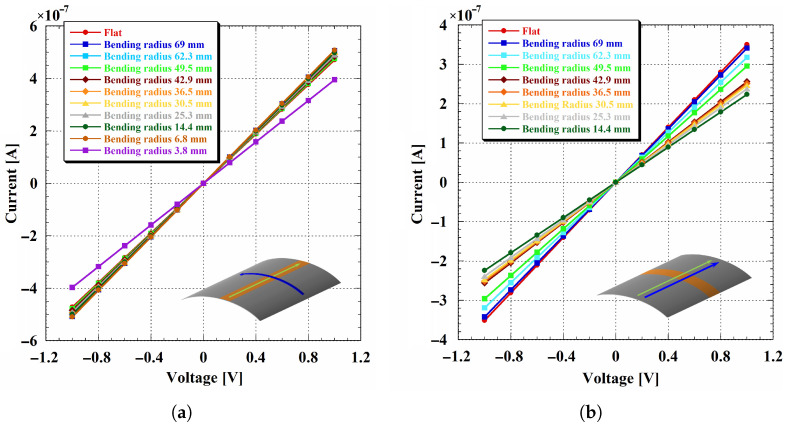
n-dopeda-Si:H layer IV measurements: (**a**) transverse configuration; (**b**) longitudinal one.

As noticeable in Figure 4a, in the transverse configuration the IV characteristics remained nearly unchanged. A slight reduction in current occurred only for the 3.8 mm bending radius, where the current shifted from a maximum value of about 0.5 µA to 0.4 µA. This behavior suggests the onset of strain-induced effects impacting the bulk conductivity of the film. Although modest, the reduction may indicate the presence of microstructural changes or an increase in series resistance induced by strong bending.

Overall, the transverse configuration appeared to be more robust against bending-induced degradation than the longitudinal one (Figure 4b). In the latter case, the device showed a more pronounced current reduction as the bending radius decreased, likely due to a more uniform strain distribution along the current path.

Indeed, in the longitudinal configuration, the current progressively decreased with bending, down to a minimum value of R8 = 14.4 mm, suggesting an increase in resistivity under mechanical strain. Bending radii smaller than R8 were not investigated, as the overall specimen geometry (including contact pads and free-end length) together with the mounting approach did not ensure a stable and repeatable curvature without overlap or slippage.

The behavior in the longitudinal configuration contrasts with the weak degradation observed in the transverse configuration, indicating a higher sensitivity of the device to bending when the current flow is aligned with the strain axis. This comparison highlights an anisotropic response of the n-doped a-Si:H layer to mechanical stress, underlying the importance of current direction in the characterization of flexible devices.

A linear fitting of the measurements was then carried out to extract the film conductance *G* and, consequently, the resistivity ρ, using the relation

(1) $$R=\frac{1}{G}=\rho \cdot \frac{L}{W\cdot H}$$

where *L* is the a-Si:H layer length, *W* is the width, and *H* is the thickness of the film.

Since the test structures were measured in a two-terminal configuration, the extracted resistivity should be regarded as an effective value, which may include a series contribution from the metal/a-Si:H contacts. However, due to the long conduction path and large contact area, the film resistance is the dominant factor in the measured area. Therefore, the reported trends can be considered representative of the deformation-induced electrical behavior of the deposited layers.

**Figure 5:**
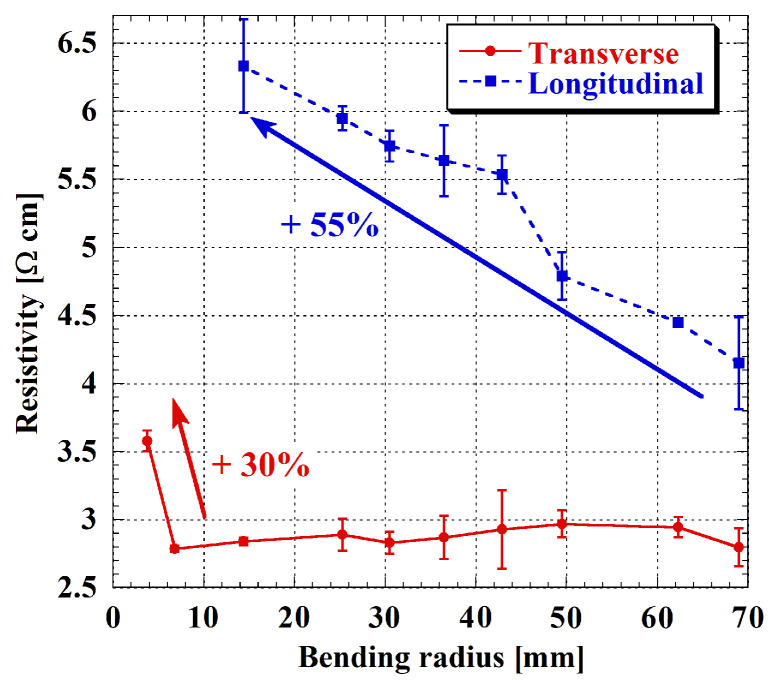
n-type a-Si:H resistivity as a function of the bending radius. Transverse configuration in red, and longitudinal one in blue. Error bars refer to measurements on the three samples under test.

As visible in Figure 5, the resistivity remained stable throughout most of the investigated bending range in the transverse configuration, with an overall increase of 30% at the smallest radius. This result suggests that the material preserves good electrical integrity under mild to moderate strain, and that failure occurs only at severe bending conditions. In contrast, the longitudinal configuration exhibited more gradual changes in resistivity, with a total increase of about 55% compared to the unbent state; furthermore, this response was entirely irreversible across all bending conditions, highlighting that stress applied along the current path more severely impacts the film’s structural and electronic integrity.

#### 3.1.2. p-Type a-Si:H Layer

**Figure 6:**
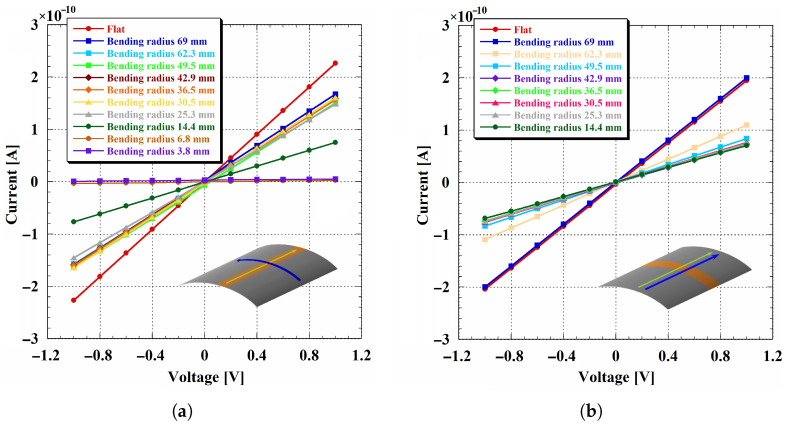
p-dopeda-Si:H layer IV measurements: (**a**) transverse configuration; (**b**) longitudinal one.

The p-type layer exhibited greater sensitivity to mechanical deformation than the n-type layer. Figure 6 shows the IV characteristics across all the bending radii in both transverse and longitudinal directions.

In the transverse configuration (Figure 6a), the IV response was more affected by bending compared to the n-type layer. Specifically, an initial reduction in current was observed during the transition from the flat state to the first bent state. This initial degradation stabilized at intermediate radii, whereas a second and more pronounced current drop occurred at the smallest radius (14.4 mm), indicating a significant deterioration of the film conductivity. This two-step evolution suggests the coexistence of an initial strain-induced modification (already triggered at mild curvature) and a critical-radius regime where damage accumulation becomes dominant, leading to a rapid loss of conductivity.

In contrast, in the longitudinal configuration (Figure 6b), the first significant change in the IV characteristics was observed between 69 mm and 62.3 mm. No substantial changes were observed at smaller radii, suggesting a weaker dependence of mechanical deformation when the current flows parallel to the bending axis. This behavior is consistent with a progressive strain accommodation, where the dominant electrical change occurs at the first curvature step, followed by a comparatively stable response over the subsequent radii explored.

**Figure 7:**
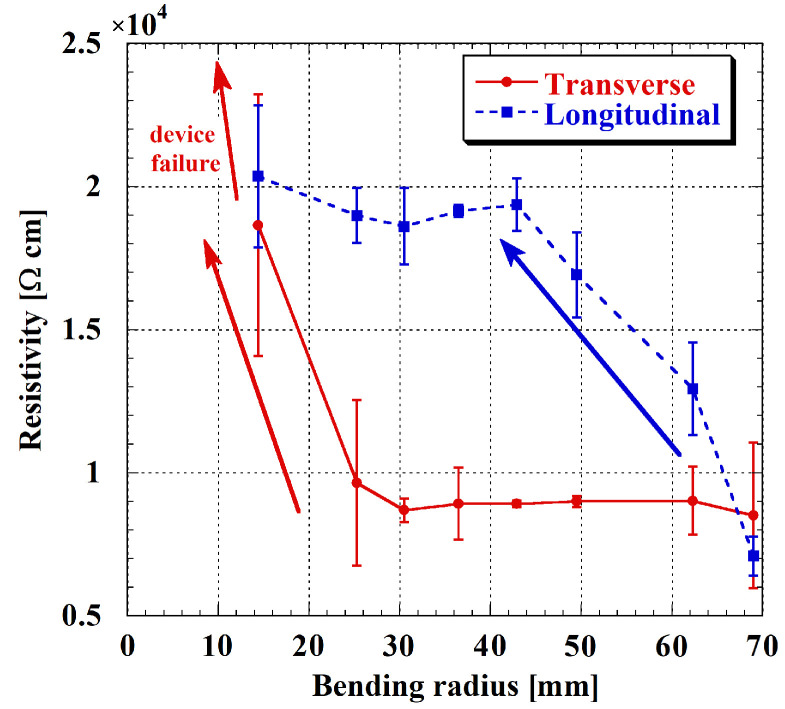
p-type a-Si:H resistivity as a function of the bending radius. Transverse configuration in red, and longitudinal one in blue. Error bars refer to measurements on the three samples under test.

Subsequently, resistivity values were calculated (Figure 7). In the transverse direction, the resistivity remained almost constant for bending radii between 69 mm and 30.5 mm. Below this threshold, a sharp increase in resistivity was observed, reaching values of tens of kilo-ohms at bending radius of 14.4 mm. At the smallest curvature radii (6.8 and 3.8 mm), resistivity reached hundreds of mega-ohms (not reported in the graph), indicating electrical failure of the film. This confirms a threshold-like behavior, where the electrical response remains stable up to moderate strain and then rapidly degrades once a critical curvature is exceeded. As for the n-type layer, significant degradation occurred only beyond a critical bending radius, while resistivity, and thus electrical properties, remained nearly unchanged at larger radii.

In the longitudinal configuration, the degradation was more gradual. Complete failure was not observed within the investigated range, likely due to geometric constraints that prevented testing at smaller bending radii. The resistivity increased gradually as the radius decreased, reaching a value approximately three times higher than the initial one, and then remained stable for all radii below 42.9 mm.

#### 3.1.3. Directional Comparison

A direct comparison of the two bending configurations reveals similar, albeit different, trends for the n- and p-doped layers. In the transverse direction, both materials initially exhibited stable resistivity as bending increased, with no significant degradation until a critical radius was reached. For the n-doped layer, this transition resulted in a slight increase in resistivity without device failure. In contrast, the p-doped layer showed a much stronger degradation, ultimately leading to electrical failure at the smallest bending radius. Despite this difference in magnitude, resistivity remained nearly constant for both layers at larger radii, indicating good tolerance to moderate transverse mechanical stress.

In the longitudinal direction, stress effects were immediately evident at the largest radii investigated and progressively increased with decreasing radius. In the n-doped layer, resistivity rose steadily over the entire bending range, although the overall variation remained limited (approximately +55%). The p-type layer instead exhibited a substantially larger resistivity increase, reaching nearly three times the initial value and stabilizing at radii smaller than 42.9 mm. These results suggest that longitudinal bending causes continuous deformation along the current path, whose cumulative effects largely depend on the mechanical strength of the doped layer.

The different trends observed in the two configurations can be qualitatively explained by the orientation of the micro-cracks formed during bending relatively to the current path. If the cracks are predominantly perpendicular to the current flow, their influence is negligible as long as they are few and short. However, as they propagate and increase in number, they can gradually disrupt the conduction channel, leading to a rapid increase in the resistance. This results in a threshold-like behavior with rapid deterioration beyond a critical bending radius. On the other hand, if the cracks are more aligned with the current direction, they would not immediately interrupt the current path but rather reduce the effective conductive cross-section gradually, while the current redistributes through the remaining intact regions. In this case, resistivity is expected to change more gradually with bending, which is consistent with the more uniform trends observed in the longitudinal configuration.

Moreover, all observed degradations were permanent, which is consistent with irreversible modifications in the a-Si:H films and/or in the interfaces with the substrate once critical curvature thresholds are exceeded.

### 3.2. Performance of p-i-n Diodes Under Mechanical Bending

After characterizing the behavior of the individual doped layers, the electrical response of the completed p-i-n diodes under mechanical bending was studied. Among the 32 diodes available on each array, a subset of nine devices per sample was selected for the bending experiments, since not all diodes exhibited comparable electrical performance and some were non-functional. Non-functional devices typically exhibited either open-circuit behavior or abnormally high reverse leakage, consistent with local fabrication non-idealities (e.g., defects/pinholes in the stack or contact/via discontinuities). The same subset was measured at all bending radii for each substrate thickness in order to calculate the standard deviation over this fixed subset.

**Figure 8:**
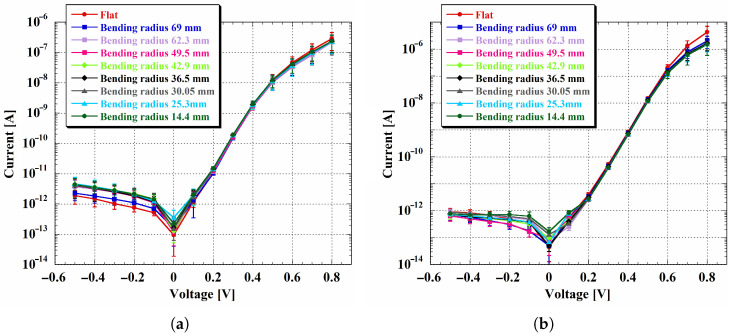
IV characteristics of p-i-n diodes fabricated on 25 (**a**) and 50 (**b**) µm thick Kapton^®^ substrates, measured in the flat condition and under static mechanical bending. In both graphs, error bars represent the standard deviation calculated over the nine selected devices.

As shown in Figure 8, the devices exhibited stable operation for both Kapton^®^ thicknesses down to the smallest bending radius tested. In all cases, the IV characteristics maintained their rectifying behavior under static bending, indicating that the a-Si:H multilayer structure retains its electrical functionality even under significant mechanical deformation. In particular, the reverse current at −0.5 V remained consistent across the investigated radii, confirming that diode operation was not compromised down to a radius of 14.4 mm. This apparent robustness, compared to the stronger strain sensitivity observed in the isolated doped layers, can be explained by the p-i-n device geometry: the dominant current path is perpendicular to the substrate surface and is therefore less directly disrupted by strain-induced features developing along the bent surface. As a result, the multilayer junction preserves rectification even when single-layer resistive structures exhibit higher in-plane sensitivity.

A closer inspection revealed subtle differences between devices fabricated on 25 and 50 µm thick Kapton^®^ substrates. In particular, diodes on the thinner substrate exhibited a slightly higher reverse current already in the flat state, as well as a small but measurable increase (on the order of a few picoamps) during the initial bending stages. Moreover, in hydrogenated amorphous silicon, an exponential increase in reverse current is commonly observed and attributed to defect-assisted transport mechanisms [96]. The steeper reverse-current slope observed for the thinner substrate may therefore indicate a substrate-thickness-dependent effect on the a-Si:H film quality introduced during fabrication, potentially related to differences in stress relaxation or thermal coupling during film growth. This interpretation is further supported by the earlier onset of the high-injection regime under forward bias observed in devices fabricated on the same substrate. This is consistent with a higher defect density that leads to a reduction in effective doping in a-Si:H p-i-n junctions [50].

Despite these initial differences, the reverse current of devices fabricated on the 25 µm thick substrate stabilized as the bending radius decreased, with no evidence of progressive degradation. Devices fabricated on 50 µm thick Kapton^®^ exhibited a similarly stable reverse-current behavior starting from the flat condition, indicating that mechanical bending does not introduce additional degradation beyond the variations established during fabrication.

Overall, these results suggest that, although thinner substrates may exhibit slightly larger and steeper reverse currents, the p-i-n architecture effectively ensures stable electrical operation under bending. Compared to single-layer structures, the multilayer junction shows enhanced mechanical robustness, supporting the integration of p-i-n a-Si:H devices on flexible substrates for sensor platforms operating in curved or portable environments.

## 4. Conclusions

This work investigated the mechanical robustness of hydrogenated amorphous silicon thin-film structures deposited on polyimide substrates (Kapton^®^), with the aim of enabling their integration into flexible sensor platforms. The study was intentionally structured from material-level characterization (single-doped layers) to device-level validation (p-i-n junctions), thereby establishing a direct link between stress orientation, degradation thresholds, and overall diode robustness.

Measurements on single layers revealed a clear dependence of electrical behavior on the direction of mechanical deformation. Under transverse bending, both n- and p-doped layers exhibited stable resistance over a wide range of radii, with degradation occurring only beyond a critical bending threshold. However, the severity of degradation was strongly doping-dependent. Indeed, the n-doped layer maintained its electrical integrity and showed only moderate resistance variations, whereas the p-doped layer exhibited a sharp resistance increase at small bending radii, ultimately leading to electrical failure. In contrast, longitudinal bending induced degradation at higher radii values, which progressed continuously as the bending increased. This behavior confirms that strain applied along the current path is more detrimental and that bending/release cycles lead to irreversible changes.

In complete p-i-n diodes, the multilayer junctions maintained their rectifying behavior even at the smallest bending radius tested (14.4 mm), for both 25 and 50 µm thick Kapton^®^ substrates. Therefore, this demonstrates that the device architecture remains electrically functional even under significant static deformation. Furthermore, differences between the two substrates’ thicknesses were already evident in the flat state: devices on the thinner substrate showed a steeper reverse-current slope and an earlier onset of high-injection behavior. This suggests that substrate thickness influences film quality and the effective doping achieved during fabrication, and that these effects did not arise from the progressive degradation caused by bending. Notably, no additional cumulative degradation was observed upon bending, highlighting the superior mechanical robustness of the p-i-n configuration compared to single-layer films. This enhanced robustness is likely due to the current flow direction in p-i-n diodes, which is perpendicular to the bent surface.

Overall, these results confirm the feasibility of integrating p-i-n a-Si:H devices on polyimide substrates for flexible sensor platforms on curved or wearable surfaces. However, the present study focuses on controlled static curvature as a quantitative benchmark; therefore, the results do not yet address long-term reliability under cyclic/fatigue bending nor combined environmental stressors beyond the controlled laboratory conditions adopted here. In addition, the smallest accessible radii for some configurations are limited by practical constraints of specimen geometry and mounting, which restrict the range of stable and repeatable curvature conditions.

Future work will extend the present static-curvature benchmark by including cyclic/fatigue bending tests and combined environmental–electromechanical stress (temperature/humidity excursions and cycling) to assess long-term stability under wearable-relevant conditions. In parallel, we will investigate encapsulation and contact/interface engineering strategies and will evaluate application-oriented sensor metrics (e.g., sensitivity, repeatability, and drift) while the devices operate under curvature.

## Figures and Tables

**Table 1:** Qualitative comparison between the proposed a-Si:H devices on Kapton^®^ and representative thin-film technologies for flexible sensors.

Technology/ Device Family	Process Compatibility with Polymers	Key Advantages for Flexible Sensors	Main Drawbacks/ Limitations	Typical Use Cases (Examples)
a-Si:H p-i-n junctions and TFT [this work] [56,66]	Low-temperature PECVD; large-area thin-film deposition compatible with polymer substrates	Mature inorganic platform; good electrical functionality under static bending; scalable thin-film process	Degradation over time due to the Staebler-Wronski effect (light-induced defects); low carrier mobility; potential mechanical cracking	Flexible thin-film sensor stacks; wearable physiological patches and sensor front-ends (TFT readout on flexible foils)
Organic semiconductors (e.g., OFET-based sensors) [81,82,83]	Solution/ low-temperature processing; highly polymer-friendly; large-area deposition	High compliance; lightweight; potentially low-cost manufacturing	Lower electronic performance; environmental sensitivity and drift; reproducibility can be process-dependent	Wearable bioelectronics; soft chemical/physical sensors; low-power flexible electronics
Oxide semiconductors (e.g., IGZO- or In_2_O_3_-based TFTs/ devices) [84,85,86]	Sputtering/ALD; compatible with flexible substrates using low-temperature flows	Good electrical performance; scalable manufacturing; established ecosystem	Process complexity; performance shifts under strain; anneal constraints may apply	Flexible backplanes; integrated sensing + electronics; transparent/flexible devices
2D materials(e.g., graphene, MoS_2_) [87,88,89]	Transfer/growth/ integration vary; polymer integration feasible but process-dependent	Very thin and flexible; high surface sensitivity; wide material choices	Large-area uniformity and reproducibility; contact variability; graphene has no bandgap	Strain/pressure sensors; gas/chemical sensors; flexible sensing skins
Silicon nanomembranes/ thin crystalline silicon on flexible substrates [90,91,92]	Transfer printing/thinning + bonding; more complex integration than thin films	High electronic performance; CMOS-grade materials and design leverage	Complex fabrication and integration; higher cost; robustness often relies on structural layouts	High-performance flexible electronics and sensors; CMOS-like functions on flexible substrates
Printed nanomaterial composites (e.g., CNT/graphene inks) [93,94,95]	Printing/low-temperature; excellent polymer/textile compatibility; large-area scalable	Low-cost and conformal; easy integration; high stretchability possible	Variability, hysteresis, and drift; calibration and long-term stability often critical	E-textiles; strain/pressure/ humidity sensors; large-area wearable sensing

**Table 2:** Cylindrical supports used for the static bending tests. The corresponding first-order estimate of the tensile strain at the top Kapton^®^ surface is reported for 25 and 50 µm thick substrates, computed as ε≈t/(2R).

Bending Radius	*R* [mm]	ε [%]@25 µm	ε [%]@50 µm
R1	69	0.018	0.036
R2	62.3	0.020	0.040
R3	49.5	0.025	0.051
R4	42.9	0.029	0.058
R5	36.5	0.034	0.068
R6	30.5	0.041	0.082
R7	25.3	0.049	0.099
R8	14.4	0.087	0.174
R9	6.8	0.184	0.368
R10	3.8	0.329	0.658

## Data Availability

The original contributions presented in the study are included in the article. Further inquiries can be directed to the corresponding authors.

## Abbreviations

The following abbreviations are used in this manuscript:

ALD	Atomic layer deposition
a-Si:H	Hydrogenated amorphous silicon
Al	Aluminum
Cr	Chromium
CMOS	Complementary metal–oxide–semiconductor
CNT	Carbon nanotube
CTE	Coefficient of thermal expansion
IGZO	Indium gallium zinc oxide
In_2_O_3_	Indium oxide
IV	Current–voltage
MoS_2_	Molybdenum disulfide
PECVD	Plasma-Enhanced Chemical Vapor Deposition
PEN	Polyethylene naphthalate
PET	Polyethylene terephthalate
PI	Polyimide
p-i-n	p/intrinsic/n junction
SU-8	Negative photoresist (SU-8)
TFT	Thin-Film Transistor
Tg	Glass transition temperature
Ti-W	Titanium–tungsten alloy
